# Effects of a Ruthenium Schiff Base Complex on Glucose Homeostasis in Diet-Induced Pre-Diabetic Rats

**DOI:** 10.3390/molecules23071721

**Published:** 2018-07-14

**Authors:** Lindokuhle Patience Mabuza, Mlindeli Wilkinson Gamede, Sanam Maikoo, Irvin Noel Booysen, Phikelelani Siphosethu Ngubane, Andile Khathi

**Affiliations:** 1 School of Laboratory Medicine and Medical Sciences, College of Health Sciences, University of KwaZulu-Natal, Durban 4000, South Africa; 211509843@stu.ukzn.ac.za (L.P.M.); 213571877@stu.ukzn.ac.za (M.W.G.); Ngubanep1@ukzn.ac.za (P.S.N.); 2 School of Chemistry and Physics, College of Agricultural and Environmental Sciences, University of KwaZulu-Natal, Pietermaritzburg 3209, South Africa; 208501744@stu.ukzn.ac.za (S.M.); booyseni@ukzn.ac.za (I.N.B.)

**Keywords:** pre-diabetes, ruthenium complex, glycated haemoglobin, dietary intervention

## Abstract

Pre-diabetes is a condition that precedes type 2 diabetes mellitus (T2DM) that is characterised by elevated glycated haemoglobin (HbA1c). The management of pre-diabetes includes the combination of dietary and pharmacological interventions to increase insulin sensitivity. However, poor patient compliance has been reported with regard to dietary interventions, therefore, new alternative drugs are required that can be effective even without the dietary intervention. In our laboratory, we have synthesised a novel ruthenium complex that has been shown to have elevated biological activity. This study investigated the effects of this complex in both the presence and absence of dietary intervention on glucose handling in a diet-induced pre-diabetes rat model. Pre-diabetic animals were randomly assigned to respective treatment groups. The ruthenium complex was administered to pre-diabetic rats once a day every third day for 12 weeks. The administration of the ruthenium complex resulted in reduced fasting blood glucose, food intake, and body weight gain which was associated with decreased plasma ghrelin, insulin, and HbA1c levels in both the presence and absence of dietary intervention. The administration of the ruthenium complex ameliorated glycaemic control and insulin sensitivity in pre-diabetic rats. The results of this study warrant further investigations as this compound could potentially be able to re-sensitize insulin resistant cells and reduce the incidence of T2DM.

## 1. Introduction

According to the American Diabetes Association (ADA), pre-diabetes is defined as impaired fasting glucose (IFG), impaired glucose tolerance (IGT) and elevated glycated haemoglobin (HbA1c) [1,2]. The measurement of glycated haemoglobin (HbA1c) has been the most widely used test for monitoring glycaemic control in individuals with pre-diabetes [2]. In pre-diabetes, higher amounts of HbA1c indicate poor control of blood glucose levels [1,2]. The regulation of blood glucose levels is a highly integrated process involving the balance of various hormones [3]. Impaired insulin action is identified in the pathophysiology of pre-diabetic abnormalities in glucose, lipid, and protein metabolism [4]. Additionally, the meal-induced decrease of ghrelin levels is impaired in pre-diabetic patients due to lack of insulin effects [5,6,7]. These pre-diabetic abnormalities lead to polyphagia and the subsequent progression to type 2 diabetes [8,9,10]. 

The combination of pharmacological and dietary interventions is central in preventing the progression of pre-diabetes to type 2 diabetes mellitus (T2DM) [11,12]. However, patients often become over-dependent on the pharmacological treatment and neglect the dietary modifications [11]. This places the patients at a greater risk for developing T2DM [11]. Therefore, alternative treatments are needed that will remain effective even in the absence of dietary modifications. Recent literature has shown significant progress in the utilization of transition metal complexes as metal-based drugs to manage diabetes [13]. Antonyan et al. (2014) demonstrated that the coordination of ruthenium to the natural product, curcumin and the resultant ruthenium(II)-curcumin complex is promising for the development of dipeptidyl peptidase-4 (DPP-IV) inhibitors [14]. Furthermore, this ruthenium complex has been shown to possess anti-diabetic properties through anti-inflammatory, anti-oxidant, and vasodilative mechanisms [14,15]. In our laboratory, we have synthesised a ruthenium Schiff base complex that has been reported to possess increased biological activity including anti-bacterial and anti-oxidant activity as well as DNA binding capability [16]. However, the effects of this novel compound on glucose handling in the pre-diabetic state remain unknown. Hence, this study sought to investigate the effects of the ruthenium Schiff base complex on glucose homeostasis in the presence and absence of dietary intervention in a diet-induced pre-diabetic rat model.

## 2. Results

### 2.1. Caloric Intake 

By comparison with the normal control group, the pre-diabetic control group showed a significant increase in caloric intake throughout the treatment period (*p* < 0.05; Table 1). Administration of ruthenium and high fat high carbohydrate diet resulted in a significant decrease in caloric intake throughout the treatment period by comparison to the pre-diabetic control (Table 1; *p* < 0.05). Furthermore, administration of ruthenium and dietary intervention resulted in a reduction in caloric intake to within the range of the normal control, similar results were observed in metformin-treated groups when compared to pre-diabetic control (*p* < 0.05; Table 1).

### 2.2. Body Weight Change

**Figure 1:**
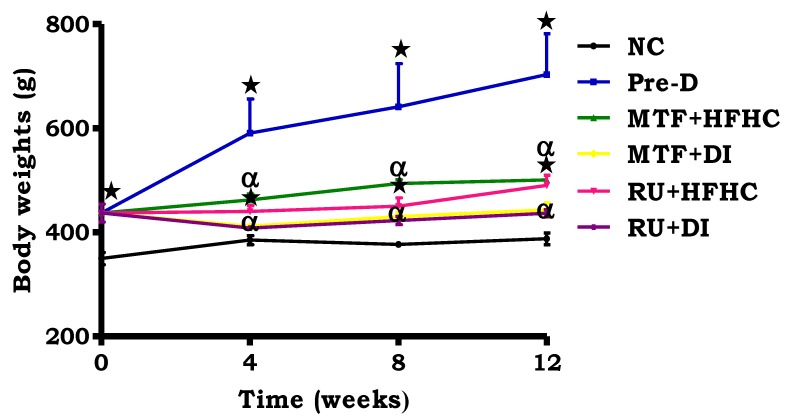
The effects of the ruthenium complex on body weight of pre-diabetic animals during treatment period. Values are presented as means ± SEM (*n* = 6) in each group. * *p* < 0.05 by comparison with normal control (NC), **^α^** *p* < 0.05 by comparison with pre-diabetic control (Pre-D); Metformin and high fat high carbohydrate (MTF + HFHC); Metformin and diet intervention (MTF + DI); Ruthenium and high fat high carbohydrate (RU + MTF); and Ruthenium and diet intervention (RU + DI).

By comparison with the NC, there was a significant increase in body weight in the pre-D control group throughout the treatment period (*p* < 0.05; Figure 1). The administration of RU + HFHC significantly reduced the rate of body weight gain by comparison to the pre-D control group (*p* < 0.05; Figure 1). Furthermore, the administration of administration of RU + DI resulted in a reduction in body weight gain to within the range of the normal control. In addition, the same effect was observed in the metformin treated groups as compared to pre-D control (*p* < 0.05; Figure 1).

### 2.3. Plasma Ghrelin Concentration

**Figure 2:**
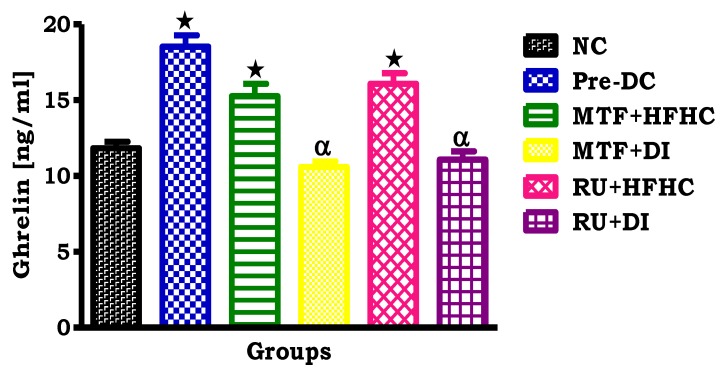
The effects of the ruthenium complex on ghrelin concentration of pre-diabetic animals during the treatment period. Values are presented as means ± SEM (*n* = 6) in each group. * *p* < 0.05 by comparison with normal control (NC), **^α^** *p* < 0.05 by comparison with pre-diabetic control (Pre-D); Metformin and high fat high carbohydrate (MTF + HFHC); Metformin and diet intervention (MTF + DI); Ruthenium and high fat high carbohydrate (RU + MTF); and Ruthenium and diet intervention (RU + DI).

By comparison with the NC group, the pre-D control group showed a significant increase in plasma ghrelin concentrations (*p* < 0.05; Figure 2). Interestingly, in comparison with the pre-D control group, the administration of both RU + HFHC and RU + DI resulted in a significant decrease in plasma ghrelin concentration (*p* < 0.05; Figure 2). The same effect was observed in the metformin treated groups when compared to Pre-D control (*p* < 0.05, Figure 2).

### 2.4. OGT Response

**Figure 3:**
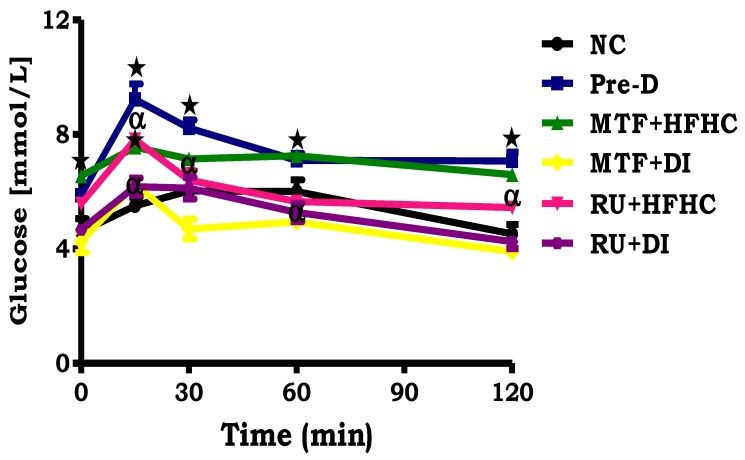
The effects of the ruthenium complex on OGTT of pre-diabetic animals. Values are presented as means ± SEM (*n* = 6) in each group. * *p* < 0.05 by comparison with normal control (NC), **^α^** *p* < 0.05 by comparison with pre-diabetic control (Pre-D); Metformin and high fat high carbohydrate (MTF + HFHC); Metformin and diet intervention (MTF + DI); Ruthenium and high fat high carbohydrate (RU + MTF); and Ruthenium and diet intervention (RU + DI).

By comparison with the NC group, the pre-D control showed a significantly increased fasting blood glucose concentration at time 0 before loading with glucose (*p* < 0.05; Figure 3). Following glucose loading, the blood glucose concentration of pre-D remained higher at all time intervals during the OGT response test as compared to the NC group (*p* < 0.05; Figure 3). Interestingly, animals who had been treated with the ruthenium complex had fasting glucose concentrations in range with those of the NC group. Additionally, the same trend was shown by the MTF + DI group when compared to pre-D control group (*p* < 0.05; Figure 3). 

### 2.5. Gastrocnemius Muscle Glycogen Concentration

By comparison with the NC group, the pre-D control group showed a significant increase in gastrocnemius muscle glycogen concentration (*p* < 0.05; Table 2). The administration of RU + HFHC resulted in a significant decrease in gastrocnemius muscle glycogen concentration by comparison to the pre-D control group (*p* < 0.05; Table 2). Additionally, the administration of RU + DI resulted in reduced glycogen levels to within the range of the NC group. A similar effect was observed in the metformin treated groups when compared to the Pre-D control group (*p* < 0.05; Table 2). 

### 2.6. HOMA2-IR Index

The HOMA2-IR value of the NC group was within the insulin-sensitivity range (< 1.9), while it was significantly increased to the moderate insulin resistance range (> 2.9) in the pre-D group (*p* < 0.05; Table 3). The administration of RU + DI resulted in a significantly decreased HOMA2-IR value to within the insulin sensitivity range (< 1.9) (*p* < 0.05; Table 3). A similar effect was observed in the MTF + DI group with significantly decreased HOMA2-IR value to within the insulin sensitivity range (< 1.9) (*p* < 0.05; Table 3).

### 2.7. Glycated Haemoglobin (HbA1c) Concentration

**Figure 4:**
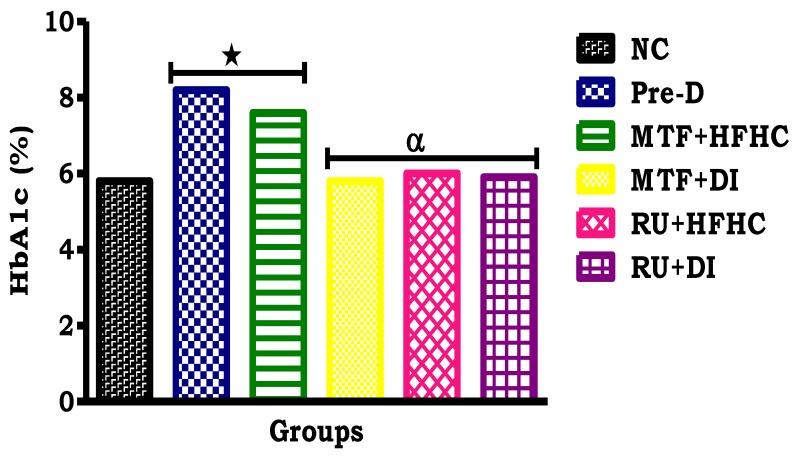
The effects of the ruthenium complex on terminal HbA1c concentration of pre-diabetic animals during treatment period. Values are presented as means (*n* = 6) in each group. * *p* < 0.05 by comparison with normal control (NC), ^α^ *p* < 0.05 by comparison with pre-diabetic control (Pre-D); Metformin and high fat high carbohydrate (MTF + HFHC); Metformin and diet intervention (MTF + DI); Ruthenium and high fat high carbohydrate (RU + MTF); and Ruthenium and diet intervention (RU + DI).

The HbAc1 concentration of the NC group was within the normal range (< 5.7%), while the pre-D significantly increased to diabetic range (> 6.5%) (*p* < 0.05; Table 3). Interestingly, by comparison with the pre-D control group, administration of the ruthenium complex showed a significant decrease in HbA1c concentration to within the normal range (< 5.7%) (*p* < 0.05; Figure 4). The same effect was observed with the MTF + DI group when compared to the Pre-D control group (*p* < 0.05; Figure 4).

## 3. Discussion

Pre-diabetes is a long-lasting condition which often precedes the onset of T2DM and is characterized by elevated levels of HbAc1 [17,18]. Pre-diabetes has been identified as a therapeutic target in the prevention of the onset of T2DM, thus reducing the incidence of diabetes-related complications [17,18]. Lifestyle interventions are central in the management of pre-diabetes [11,19]. Nutrition therapy for pre-diabetic patients is one of the lifestyle changes that have been shown to improve glycaemic control [12,20]. Since many pre-diabetic patients are insulin resistant and overweight, nutrition therapy often begins with lifestyle strategies that reduce energy intake and increase energy expenditure through dietary intervention and physical activity [21]. However, since dietary interventions are not always feasible, especially in developing communities, the combination of pharmacological and dietary interventions is implemented [11,20]. The efficacy of these pharmacological interventions often depends on reduced caloric intake, thus there is reduced efficacy in the presence of high calorie diets [20]. Therefore, there is a need to develop alternative drugs that will be able to prevent the progression of pre-diabetes to T2DM in both the presence and absence of dietary interventions. Ruthenium complexes have been shown to possess anti-diabetic properties through anti-inflammatory, anti-oxidant and vasodilative mechanisms [14,15]. In this study, we investigated the effects of the administration of the ruthenium Schiff base complex on glucose homeostasis in the presence and absence of dietary intervention in a diet-induced pre-diabetic rat model [22,23]. The present study further confirms findings from other studies that the consumption of the HFHC diet in rats significantly increased caloric intake which in turn increased body weight and body fat as compared to a standard diet [24,25]. The observed increase in body weight can be ascribed to increased caloric intake and possible accumulation of adipose tissue [22,26,27]. However, administration of the ruthenium complex along with dietary intervention reduced caloric intake suggesting that a possible mechanism of action of this complex was through the amelioration of caloric intake [26]. Furthermore, studies have shown that metformin, as a convention drug for pre-diabetes, can reduce caloric intake and hence restore body weight in obese pre-diabetic patients. These were further evidenced by the results obtained in Ref. [28]. Indeed, the ruthenium-treated animals showed decreased plasma ghrelin concentration by comparison to the pre-diabetic control group. Circulating levels of plasma ghrelin are associated with regulation of food intake and energy balance [9,29]. Insulin plays an important part in reducing postprandial ghrelin concentration [9]. In healthy individuals, plasma ghrelin levels are reduced after a meal and rise progressively before the next one [9,29]. However, in pre-diabetic patients, the meal-induced decrease in ghrelin levels is impaired, suggesting that the impaired postprandial reduction of circulating ghrelin may partly account for the glucose intolerance as well as ongoing weight gain [10]. Furthermore, researchers have shown that in patients with poorly controlled diabetes, the lack of postprandial ghrelin decreases results from a profound insulin inefficiency and may explain polyphagia [9,29]. The reduced plasma ghrelin concentrations observed in this study suggest that the ruthenium complex may restore insulin sensitivity.

Glucose tolerance disturbances in diet-induced pre-diabetic rats have been reported [23,24,25]. The administration of the ruthenium complex along with dietary intervention resulted in decreased fasting glucose concentrations as well as improved glucose tolerance suggesting increased glucose utilization by tissues. The decreased glucose concentrations can be potentially ascribed to the metal complex improving insulin sensitivity. The ability of the mononuclear metal complex to promote increased glucose utilization in the pre-diabetic treated rats may be of therapeutic value in improving glucose homeostasis in pre-diabetic patients. Sulphonylureas, such as glipizide, have been found to improve blood glucose control through increased peripheral glucose utilization [30]. Interestingly, administration of the ruthenium complex restored glycogen concentrations in skeletal muscle. These observations further suggest that this metal complex restored insulin sensitivity. The anti-diabetic activities of transition metal complexes emanate from their diverse modes of interaction with distinctive biological targets [14,31]. Other metal complexes such as the oxidovanadium(IV) and dioxidovanadium(V) compounds which are designed to be pro-drugs and convert to the active drug, vanadate, under physiological conditions have been found to have increased biological activity [32]. The active metallo-drug inhibits the protein tyrosine phosphatase (PTP) enzyme and this avoids the autophosphorylation phenomenon of insulin, ultimately culminating into the regulation of the blood glucose levels [32]. Additionally, metformin has also been shown to increase peripheral uptake of glucose and to reduce hepatic glucose output by when given orally [28].

Studies have shown that fasting insulin concentrations can be used as a marker as it is known that subjects with higher fasting insulin concentrations have higher risks of developing diabetes [33]. However, administration of the ruthenium compound in combination with dietary intervention resulted in reduced fasting plasma insulin concentrations, thus alleviating the HOMA2-IR value of pre-diabetic treated rats. These observations can be attributed to the influence of the metal complex which improves insulin sensitivity and the attenuation of hyperglycaemia in diet-induced pre-diabetic rats. HbA1c has been the most widely used and accepted test for monitoring the glycaemic control in individuals with pre-diabetes [17,34,35]. Once a haemoglobin molecule is glycated, it remains in the red blood cell for a life-span of 120 days [18,34,36]. Thus, this test provides information about the degree of long-term blood glucose control [34,36]. Administration of the mononuclear ruthenium complex showed a decreased HbA1c concentration in pre-diabetic rats. The results obtained showed that the ruthenium complex has similar effects to metformin. Although we have yet to fully establish the mechanism by which this happens, transition metal complexes have been found to have different biodistribution patterns [31]. More importantly, the anti-hyperglycaemic effects of the potential metallopharmaceutical, [Ru^II^(H_3_ucp)Cl(PPh_3_)], provides impetus to fully elucidate its mechanism of activity.

## 4. Materials and Methods

### 4.1. Chemicals and Drugs

Chemicals and drugs were sourced as follows:

Dimethyl sulphoxide (DMSO), phosphate buffered saline (PBS), anthrone, metformin, horse radish peroxidase (HRP) coupled antibodies (Sigma-Aldrich, St Louis, MO, USA); Ethanol, sodium sulphate (NaSO4), potassium hydroxide (KOH), sulphuric acid (H_2_SO_4_) (Merck chemicals, Johannesburg, South Africa); Isofor, liquid nitrogen (Safeline Pharmaceuticals (Pty) Ltd., Roodeport, South Africa); High-fat high-carbohydrate (HFHC) diet (AVI Products (Pty) Ltd., Waterfall, South Africa) and Ruthenium (II) complex, [Ru^II^(H_3_ucp)Cl(PPh_3_)] (H_4_ucp = 2,6-*bis*-((6-amino-1,3-dimethyluracilimino)methylene)pyridine) (School of Chemistry and Physics, University of KwaZulu-Natal, Pietermaritzburg, South Africa).

All other chemicals and drugs were of analytical grade purchased from standard commercial suppliers.

### 4.2. Synthesis of Ruthenium (II) Complex

The synthesis of the ruthenium(II) complex, [Ru^II^(H_3_ucp)Cl(PPh_3_)] (H_4_ucp = 2,6-*bis*-((6-amino-1,3-dimethyluracilimino)methylene)pyridine), was synthesised in our laboratory as previously reported [16]. The complex was characterized by the following conductance measurements; UV/Vis, nuclear magnetic resonance (NMR), electron spin resonance (ESR), and infrared resonance (IR) spectroscopy as well as single crystal X-ray diffraction. Previous studies have shown that the dose of the ruthenium complex used in this study was non-toxic [37].

### 4.3. Animals and Housing 

Male Sprague–Dawley rats (n = 36) weighing 150–180 g, bred and housed in the Biomedical Research Unit (BRU) of University of KwaZulu-Natal were used in this study. All animal procedures and housing conditions were approved by the Animal Research Ethics Committee of the University of KwaZulu-Natal (ethics no: AREC/038/016M). The animals had free access to food and water throughout the study period.

#### 4.3.1. Induction of Pre-Diabetes

The animals were randomly assigned to the following diet groups: standard diet with normal drinking water (NC, n = 6) and high-fat high-carbohydrate diet with drinking water supplemented with 15% fructose (HFHC n = 30) (AVI Products (Pty) Ltd., Waterfall, South Africa). Pre-diabetes was induced by allowing the animals to feed on the HFHC diet for 20 weeks as previously described [24,25]. After 20 weeks, the American Diabetes Federation criteria was used to diagnose pre-diabetes.

#### 4.3.2. Experimental Design

The study consisted of two major groups, the normal animals (NC, n = 6) and the pre-diabetic animals (n = 30). Once the animals were pre-diabetic (after 20 weeks), the pre-diabetic animals were then further subdivided into the following 5 groups: Pre-diabetic control (Pre-D), which are the pre-diabetic animals which continued with the high fat high carbohydrate (HFHC) diet; metformin and high fat high carbohydrate diet (MTF + HFHC) (Sigma-Aldrich, St Louis, Missouri, USA), which are the pre-diabetic animals that continued with the HFHC diet but received metformin during the treatment period; metformin and diet intervention group (MTF + DI), which are the pre-diabetic animals that changed to a normal diet and received metformin during the treatment period; ruthenium and high fat high carbohydrate diet (Ru + HFHC), which are the pre-diabetic animals that continued with experimental diet but received the ruthenium complex during the treatment period; ruthenium and diet intervention (Ru + DI), which are the pre-diabetic animals that changed to a normal diet and received the ruthenium complex during the treatment period.

#### 4.3.3. Treatment of Pre-Diabetic Animals

The treatment period lasted 12 weeks. The animals were treated once a day every third day at 9:00 am, where the MTF + HFHC and MTF + DI groups received an oral dose of metformin (500 mg/kg), while the Ru + HFHC and Ru + DI groups received an intramuscular injection dose of ruthenium complex (15 mg/kg). Parameters including food intake, body weights, and fasting blood glucose were monitored at weeks 0, 4, 8, and 12. Glucose tolerance was evaluated at week 20 after Pre-diabetes induction with oral glucose tolerance test (OGTT) using a previously described protocol [38]. Briefly, after an 18 h fasting period, glucose was measured (time 0) followed by loading with a glucose syrup (glucose; 0.86 g/kg, p.o.) by oral gavage using an 18-gauge gavage needle that is 38 mm long curved, with a 21/4 mm ball end (Able Scientific, Canning Vale, Australia). To measure glucose concentration, blood was collected using the tail-prick method. Glucose concentrations were measured by a OneTouch select glucometer (Lifescan, Mosta, Malta, UK). Glucose concentrations were measured at 15, 30, 60, and 120 min following glucose loading.

#### 4.3.4. Blood Collection and Tissue Harvesting

After the 12 weeks of treatment, all animals were anaesthetised with Isofor (100 mg/kg)) (Safeline Pharmaceuticals (Pty) Ltd., Roodeport, South Africa) using a gas anaesthetic chamber (Biomedical Resource Unit, UKZN, Durban, South Africa) for 3 min. Blood was collected by cardiac puncture and then injected into individual pre-cooled heparinized containers. The blood was then centrifuged (Eppendorf centrifuge 5403, Hamburg, Germany) at 4 °C, 503 g for 15 min. Plasma was collected and stored at −80 °C in a Bio Ultra freezer (Snijers Scientific, Tilburg, Holland) until ready for biochemical analysis. skeletal muscle was removed, rinsed with cold normal saline solution and snap frozen in liquid nitrogen before storage in a BioUltra freezer (Snijers Scientific, Tilburg, Netherlands) at −80 °C until biochemical analysis.

### 4.4. Biochemical Analysis

The concentration of glycated haemoglobin (HbA1c), ghrelin, and insulin were analysed in plasma using ELISA kits according to the manufacturer’s instructions (Elabscience and Biotechnology, Wuhan, China). The homeostatic model assessment (HOMA), which is used to quantify insulin resistance and beta-cell function was calculated using the HOMA calculator available at https://www.dtu.ox.ac.uk/homacalculator/.

### 4.5. Glycogen Assay

Glycogen analysis was performed in gastrocnemius muscle tissues. Glycogen assay was conducted using a well-established laboratory protocol [39]. Gastrocnemius muscle tissues (50 mg) were weighed and heated with KOH (30%, 2 mL) at 100 °C for 30 min. Thereafter, Na_2_SO_4_ (10%, 0.194 mL) was added to stop the reaction and allowed to cool. For glycogen precipitation, the cooled mixture (200 µL) was aspirated and mixed with ethanol (95%, 200 µL). The precipitated glycogen was pelleted, washed and redissolved in H_2_O (1 mL). Thereafter, anthrone (0.5 g dissolve in 250 mL of sulphuric acid, 4 mL) was added and boiled for 10 min. After cooling the absorbance was read using the Spectrostar Nano spectrophotometer (BMG Labtech, Ortenburg, Baden-Württernberg, Germany) at 620 nm. The glycogen concentrations were calculated from a glycogen standard curve.

### 4.6. Statistical Analysis

All data were expressed as mean ± standard error of mean (S.E.M.). Statistical analysis was performed using GraphPad Prism Software (version 5.00, GraphPad Software, San Diego, CA, USA). One-way analysis of variance (ANOVA) followed by the Bonferroni post hoc test was used for analysis of differences between control and experimental groups. Values of *p* < 0.05 indicate statistical significance between the compared groups.

## 5. Conclusions

In summary, we have found that the administration of the ruthenium Schiff-base complex had beneficial effects as it restored plasma insulin and ghrelin concentrations, leading to a reduction in caloric intake and body weight gain. These results were accompanied by restored glucose tolerance as well as HbA1c concentration, indicating that the metal complex restores insulin sensitivity in diet-induced pre-diabetic rats and delays the onset of T2DM in the presence and absence of dietary intervention. In addition, the optimal anti-hyperglycaemic activities of the ruthenium complex provide new scope to comprehensively delineate its mechanism of activity.

## Figures and Tables

**Table 1:** Effects of the ruthenium complex on caloric intake of pre-diabetic animals during treatment period. Values are presented as means ± SEM (*n* = 6) in each group.

Groups	Caloric Intake (kcal/g)			
	Week 0	Week 4	Week 8	Week 12
**NC**	106.18 ± 1.90	115.74 ± 2.34	123.96 ± 1.61	138.40 ± 0.87
**Pre-D**	120.72 ± 1.11 *	158.20 ± 0.64 *	184.88 ± 0.84 *	205.84 ± 0.85 *
**MTF + HFHC**	118.09 ± 0.51 *	100.54 ± 0.98 *	99.51 ± 1.52 *^α^	151.66 ± 0.69 *^,α^
**MTF + DI**	115.12 ± 0.67	102.69 ± 1.17 ^α^	120.51 ± 0.75 ^α^	144.72 ± 1.64 ^α^
**RU + HFHC**	119.17 ± 0.65 *	124.73 ± 0.31 *^,α^	155.58 ± 0.99 ^α,^*	167.44 ± 0.87 *^,α^
**RU + DI**	113.73 ± 0.98	118.94 ± 0.85 ^α^	127.09 ± 0.89 ^α^	138.82 ± 0.76 ^α^

*p < 0.05 by comparison with normal control (NC), **^α^** *p* < 0.05 by comparison with pre-diabetic control (Pre-D); Metformin and high fat high carbohydrate (MTF + HFHC); Metformin and diet intervention (MTF + DI); Ruthenium and high fat high carbohydrate (RU + MTF); and Ruthenium and diet intervention (RU + DI).

**Table 2:** Effects of the ruthenium complex on glycogen concentration of pre-diabetic animals during the treatment period. Values are presented as means ± SEM (*n* = 6) in each group.

Groups	Muscle Glycogen (nmol/g Protein)
**NC**	0.19 ± 0.03
**Pre-D**	0.73 ± 0.01 *
**MTF + HFHC**	0.43 ± 0.11 *^,α^
**MTF + DI**	0.24 ± 0.02 ^α^
**RU + HFHC**	0.31 ± 0.03 *^,α^
**RU + DI**	0.21 ± 0.01 ^α^

*p < 0.05 by comparison with normal control (NC), **^α^** *p* < 0.05 by comparison with pre-diabetic control (Pre-D); Metformin and high fat high carbohydrate (MTF + HFHC); Metformin and diet intervention (MTF + DI); Ruthenium and high fat high carbohydrate (RU + MTF); and Ruthenium and diet intervention (RU + DI).

**Table 3:** Effects of the ruthenium complex on HOMA2-IR index of pre-diabetic animals during treatment period. Values are presented as means (*n* = 6) in each group.

Groups	Plasma Glucose (mmol/L)	Plasma Insulin (mIU/L)	HOMA-IR Values
**NC**	4.60 ± 0.09	5.89 ± 0.90	1.20 ± 0.61
**Pre-D**	5.60 ± 0.32 *	20.92 ± 3.45 *	5.20 ± 2.98 *
**MTF + HFHC**	6.60 ± 0.81 *^,α^	18.00 ±3.12 *	5.30 ± 1.02 *
**MTF + DI**	4.30 ± 0.90 ^α^	7.57 ±1.32 *^,α^	1.30 ±1.15 *^,α^
**RU + HFHC**	5.60 ±1.02 *^,α^	16.17± 1.88 *^,α^	4.00 ±1.05 *
**RU + DI**	4.70 ± 0.82 ^α^	8.29 ± 2.00 ^α^	1.70 ± 0.99 ^α^

*p < 0.05 by comparison with normal control (NC), **^α^** *p* < 0.05 by comparison with pre-diabetic control (Pre-D); Metformin and high fat high carbohydrate (MTF + HFHC); Metformin and diet intervention (MTF + DI); Ruthenium and high fat high carbohydrate (RU + MTF); and Ruthenium and diet intervention (RU + DI).
